# A Stable Aluminum Tris(dithiolene) Triradical

**DOI:** 10.1021/jacs.4c05631

**Published:** 2024-05-31

**Authors:** Phuong M. Tran, Yuzhong Wang, Boris Dzikovski, Mitchell E. Lahm, Yaoming Xie, Pingrong Wei, Vladislav V. Klepov, Henry F. Schaefer, Gregory H. Robinson

**Affiliations:** † Department of Chemistry and the Center for Computational Chemistry, The University of Georgia, Athens, Georgia 30602-2556, United States; ‡ Department of Chemistry and Chemical Biology, and ACERT, National Biomedical Center for Advanced Electron Spin Resonance Technology, Cornell University, Ithaca, New York 14853-1301, United States

## Abstract

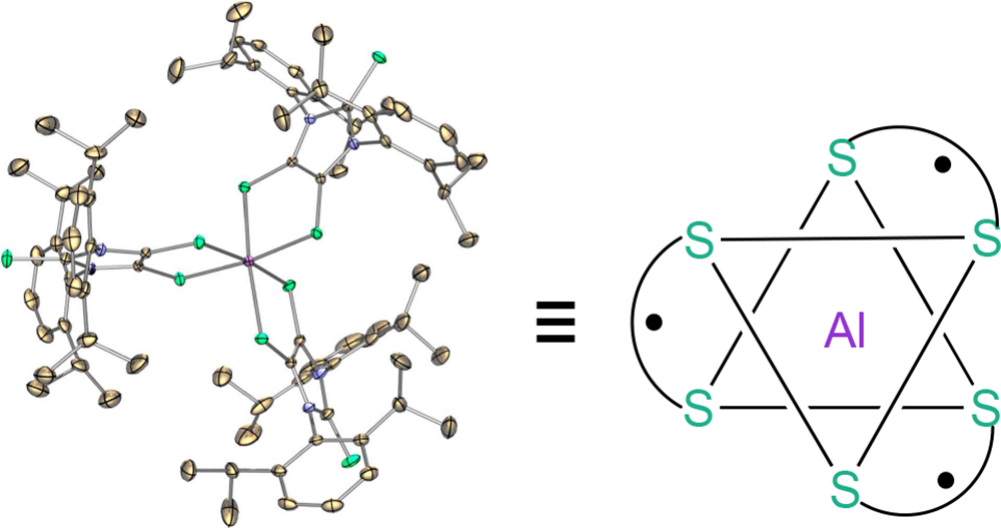

A stable aluminum tris(dithiolene) triradical (**3**) was experimentally realized through a low-temperature reaction of the sterically demanding lithium dithiolene radical (**2**) with aluminum iodide. Compound **3** was characterized by single-crystal X-ray diffraction, UV–vis and EPR spectroscopy, SQUID magnetometry, and theoretical computations. The quartet ground state of triradical **3** has been unambiguously confirmed by variable-temperature continuous wave EPR experiments and SQUID magnetometry. Both SQUID magnetometry and broken-symmetry DFT computations reveal a small doublet–quartet energy gap [Δ*E*_DQ_ = 0.18 kcal mol^–1^ (SQUID); Δ*E*_DQ_ = 0.14 kcal mol^–1^ (DFT)]. The pulsed EPR experiment (electron spin echo envelop modulation) provides further evidence for the interaction of these dithiolene-based radicals with the central aluminum nucleus of **3**.

## Introduction

**Figure 1:**
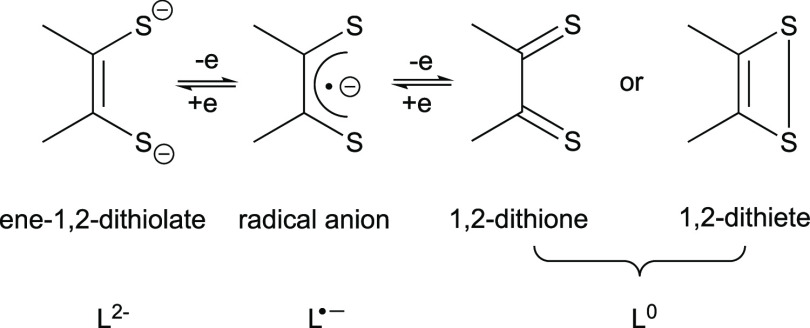
Redox states of dithiolene ligands.

The chemistry of transition-metal dithiolene complexes has been extensively explored over the past six decades.^1−11^ These complexes have fascinated chemists due to their unusual optical, conductive, magnetic properties and pivotal roles in metalloenzymes.^4−8^ The noninnocent dithiolene ligand may alter its oxidation state among ene-1,2-dithiolate dianion (L^2–^), radical monoanion (L^•–^), and neutral 1,2-dithione/1,2-dithiete (L^0^) ligand forms (Figure 1) in the corresponding transition-metal complexes,^12,13^ enabling redox changes between the transition-metal ions and the dithiolene ligand.^3^

Significant advances in the chemistry of stable organic diradicals and polyradicals, usually involving carbon, nitrogen, and oxygen elements, have been reported.^14−29^ Organic di- or polyradicals with high-spin ground states and large energy gaps (Δ*E*) between the high-spin ground state and low-spin excited state are intriguing due to their remarkable application potentials in magnetic materials.^14,15^ Notably, main group element-based polyradicals beyond carbon, nitrogen, and oxygen are scarce.^30−36^ Since the radical character of the dithiolene ligand in transition-metal complexes was first proposed by Gray et al. in the early 1960s,^37,38^ transition-metal dithiolene complexes with radical dithiolene ligands have received significant attention.^12^ Typically, tris(dithiolene)^10^ metal complexes involve three dianionic dithiolate ligands (L^2–^). Only a few tris(dithiolene) complexes containing one or two radical dithiolene ligand(s), such as [N(*n*-Bu)_4_]_2_[Cr^III^(Cl_2_-bdt^•^)(Cl_2_-bdt)_2_] (bdt = benzene-1,2-dithiolate)^39^ and [N(*n*-Bu)_4_][Cr^III^(tbbdt^•^)_2_(tbbdt)] (tbbdt = 3,5-di-*tert*-butylbenzene-1,2-dithiolate),^40^ have been isolated.^10,12^ While attempts to isolate [Cr^III^(tbbdt^•–^)_3_]^0^ were reportedly unsuccessful, this species was generated electrochemically and probed by spectroscopic and theoretical methods.^40^

The chemistry of main group element-based tris(dithiolene) complexes has not developed in parallel with that of their transition-metal counterparts.^10^ Main group tris(dithiolene) complexes have mainly involved heavier main group metals such as indium,^41−43^ thallium,^43^ tin,^44−46^ and antimony.^47−49^ Notably, structurally characterized metalloid (such as Si^50^ and Ge^51^)-based tris(dithiolene)dianions have recently been reported. The isolation of main group element-based tris(dithiolene) radicals may present unique challenges, since there are no available d orbitals to stabilize dithiolene-based π-radicals.^10^

**Scheme 1:**
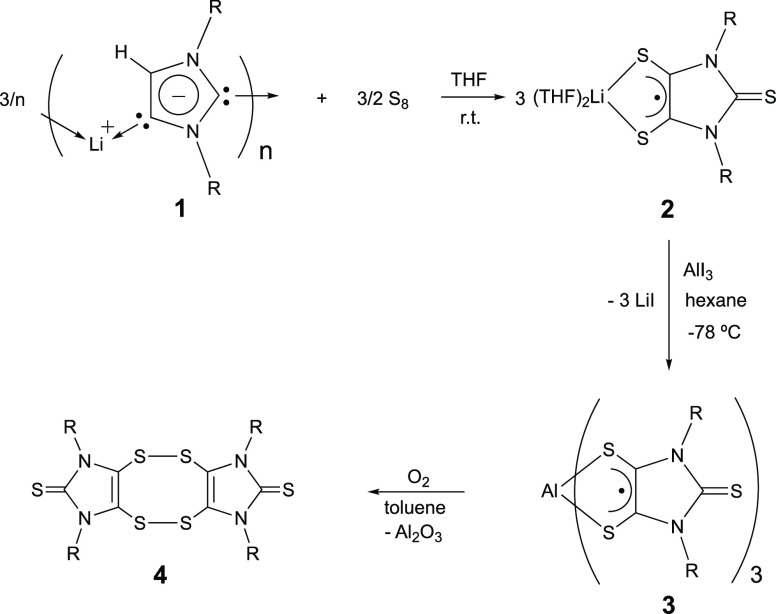
Synthesis of 3 (*R* = 2,6-diisopropylphenyl) and its Reactivity with O_2_

In contrast to the absence of tris(dithiolene) triradical species, transition-metal (such as Cr^III^)-^52^ and main group element (such as aluminum,^53−56^ gallium,^53−58^ indium,^53,55^ and silicon^59^)-based tris(dioxolene) di- and triradical species have been reported. Recently, this laboratory synthesized the first structurally characterized lithium dithiolene radical (**2**), via trisulfurization of the corresponding anionic N-heterocyclic dicarbene (NHDC) (**1**) (Scheme 1).^60^ Radical **2** has served as a unique synthetic platform to access main group element (such as magnesium^61^ and boron^62^)-based dithiolene radical species and the metal-free “naked” dithiolene radicals.^63,64^*Could radical***2***be utilized to access a main group element-based tris(dithiolene) triradical species?* Herein, we report the synthesis,^65^ molecular structure,^65^ spectral^65^ and computational^65^ studies of a stable aluminum-based tris(dithiolene) triradical (**3**), which represents the first structurally characterized tris(dithiolene) triradical complex.

## Results and Discussion

### Synthesis and UV–Vis Absorption Spectra

Triradical **3** was obtained as dark blue crystals (in 17% yield) via the 3:1 reaction of **2** with AlI_3_ in hexane (at −78 °C) and subsequent recrystallization in a toluene/hexane mixed solvent (at −40 °C) (Scheme 1).^65^ Triradical **3** is thermally unstable above 115 °C. The reaction of **3** with O_2_ (excess) in toluene gives the previously reported dithione dimer (**4**)^66^ (aluminum oxide is deduced as a byproduct, Scheme 1). In addition, in polar solvents (such as THF, CH_3_CN, and CH_2_Cl_2_), **3** immediately decomposes, giving an orange red slurry of **4**.

**Figure 2:**
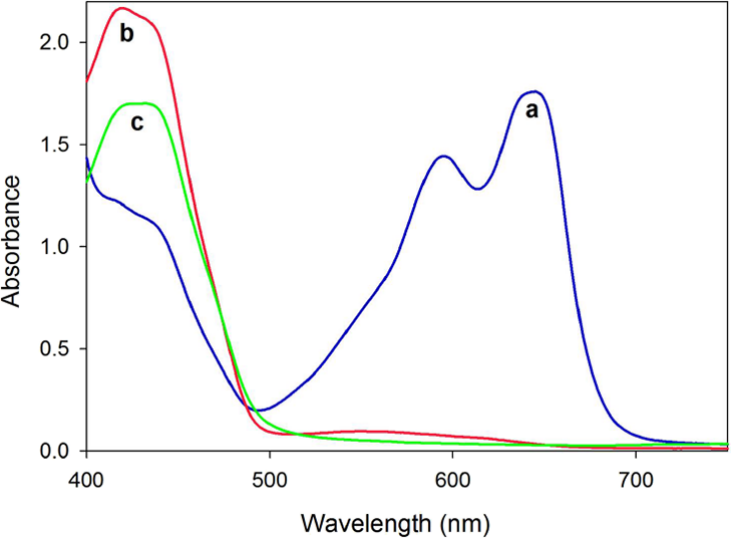
(a) UV–vis spectrum of **3** (partially oxidized during the measurement). (b) UV–vis spectrum of **3** (completely oxidized). (c) UV–vis spectrum of **4** (all spectra were measured in toluene).

Due to its extremely high sensitivity toward O_2_, **3** quickly changes color from dark blue to blue-green, even while being stored in a screw-cap cuvette (under an argon atmosphere) during the UV–vis spectral measurement. Consequently, only the UV–vis spectrum of the partially oxidized **3** was obtained, which contains four absorptions at 423, 435, 595, and 645 nm in the visible region (Figure 2a). Accompanying the complete decomposition of **3** (yellow solution), the two absorptions at 595 and 645 nm disappear, whereas the intensity of the absorption peaks at 423 and 435 nm increases (Figure 2b). The 423 and 435 nm signals are consistent with those observed in the UV–vis spectrum of **4** (Figure 2c). The TD-DFT computation (UB3LYP/6-311G**, SMD, toluene) of the **Λ-3-H** model reveals two major absorption bands at 545 [*f* (oscillator strength) = 0.02] and 594 nm (*f* = 0.43), which compare to the absorptions of **3** at 595 and 645 nm, respectively. While the absorption at 545 nm mainly involves the HOMO–4 → SOMO3 and HOMO–5 → SOMO2 electronic transitions, the absorption at 594 nm is largely due to HOMO → SOMO2 and HOMO → SOMO3 electronic transitions (Figure S11).

### Electron Paramagnetic Resonance Spectra

**Figure 3:**
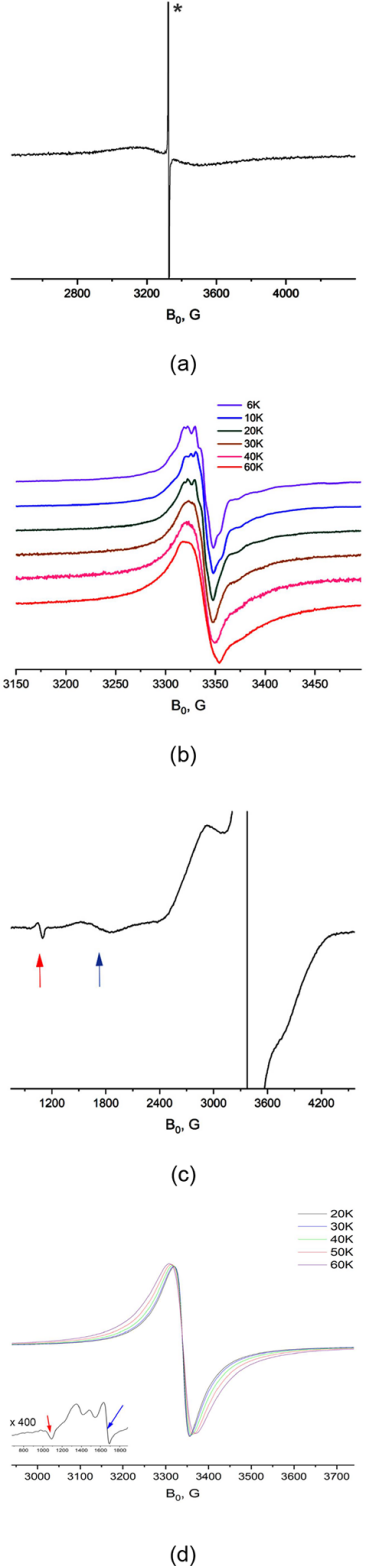
(a) Room-temperature X-band (9.4 GHz) continuous wave EPR (CW-EPR) spectrum of **3** in toluene. The intense narrow line (marked with *) is due to a monoradical admixture. (b) Main lines (Δ*m* = 1, centered at ca. 3330 G) observed in the X-band (9.4 GHz) CW-EPR spectra of **3** in toluene at different temperatures normalized by intensity. (c) Less intense features observed in the zoom of the full-scan EPR spectrum of **3** at 10 K (marked by blue and red arrows) correspond to the forbidden Δ*m* = 2 (centered at 1685 G) and Δ*m* = 3 (centered at 1080 G) transitions, respectively. (d) Solid-state X-band EPR spectra of **3** at 20–60 K. The features corresponding to the forbidden transitions at Δ*m* = 2 (centered at ca. 1669 G, blue arrow) and Δ*m* = 3 (centered at 1070 G, red arrow) are shown in the inset plot (the spectrum was measured at 20 K).

The room-temperature EPR spectrum (Figure 3a) of **3** (in toluene) only exhibits an unresolved broad singlet with a line width of ca. 360 G, which corresponds to a relaxation time T_2_ of ca. 0.15 ns. The intense narrow line (in Figure 3a) is due to a monoradical admixture. Its integral intensity is less than 0.5% of the signal.^65^ The paramagnetic properties of **3** were further probed by variable-temperature (VT) EPR spectroscopy in toluene at 6–60 K, wherein the forbidden Δ*m* = 2 and Δ*m* = 3 transitions were observed at 1685 and 1080 G, respectively (see the full scan spectrum at 10 K in Figure 3c). The presence of these transitions, with the temperature dependence of the intensity following the Curie law (see Figure S1 in the Supporting Information),^65^ supports the quartet ground state triradical character of **3**.^28,32,67^

The sharp Δ*m* = 3 transition requires all three electron spins to be coupled to each other.^68^ Moreover, the integral ratio of the Δ*m* = 1, 2, and 3 features is expected approximately to be 1:(*D*/*B*_0_)^2^: (*D*/*B*_0_)^4^, where *D* is the electron dipolar splitting, and *B*_0_ is the magnetic field corresponding to the Δ*m* = 1 resonance.^68^ Thus, for a weak dipolar coupling, the Δ*m* = 3 feature cannot be observed due to the low intensity of its EPR line. An unusual feature of **3** is the relatively strong intensity of these forbidden transitions pointing to a very large value of *D* and very close distance between interacting electrons. Estimates based on the integral intensity ratio^68^ between different allowed and forbidden transitions give *D*/*B*_0_ in a 1/9 to 1/12 range (*D* = 280–370 G). This is consistent with the total field range of the Δ*m* = 1 line (centered at ca. 3330 G), which is predicted to be about 4*D* including the smaller satellite lines spaced by 2*D* and 4*D* distances from the center. In our case, the satellite lines are not resolved but contribute to the shoulder feature in the field range of approximately 2600–4100 G (Figure 3c), since they are broadened and low in intensity compared to the main line. This is likely due to a large D strain effect (Figure S2).^65^ Based on the formula  (where *r* is the interspin distance given in Å, and *D* is in Gauss)^69^ and *g* = 2 for the Δ*m* = 1 line, the distance between the three interacting electrons can be estimated to be in the range of 4.2–4.6 Å.

**Figure 4:**
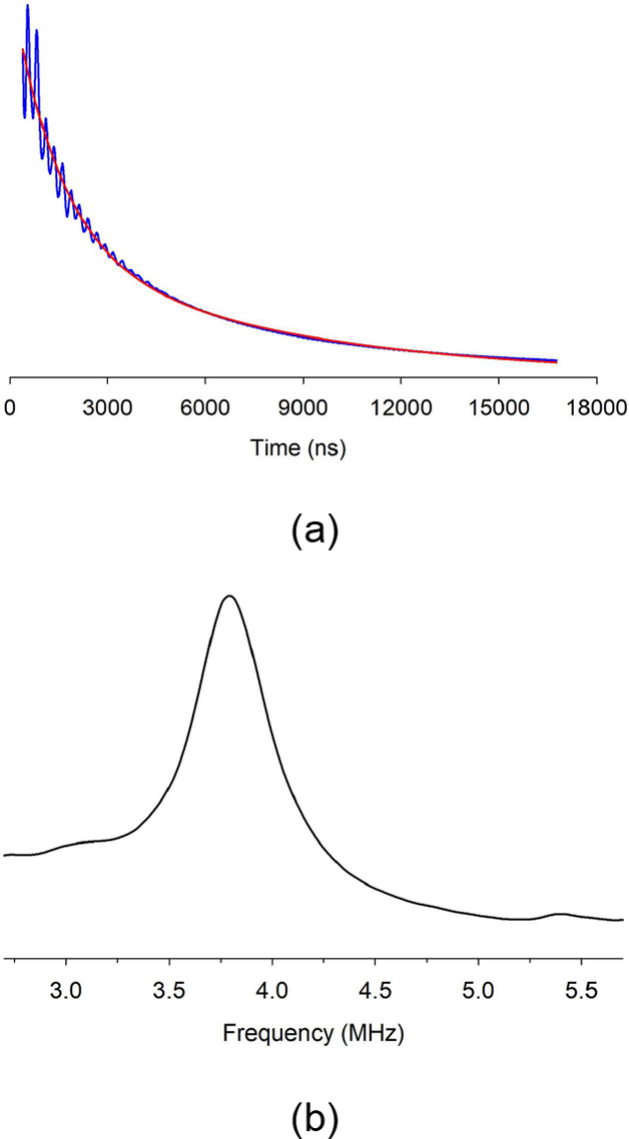
Time domain (a) and Fourier transform (b) of the three-pulse ESEEM spectra of **3** collected at 10 K. [Note: in (a), the red line represents the exponential baseline.]

The most intense signal (*g* = ca. 2.01) of the low-temperature EPR spectra of **3** corresponds to the Δ*m* = 1 transition, which shows a partially resolved hyperfine pattern with a value of ca. 5.0 G at temperatures below 20 K (Figure 3b). It should correspond to the hyperfine splitting on aluminum. For comparison, the hyperfine splitting on ^25^Mg in our previously reported magnesium-based dithiolene radical was 2.3 G.^61^ The hyperfine splitting on two equivalent ^14^N nuclei of the dithiolene ligand (*a*_N_ = ca. 1 G)^61^ cannot be resolved within this intrinsic line width. The spectrum starts to broaden and lose resolution above 20 K (Figure 3b), which can be attributed to a decrease in the T_2_ relaxation time with the increase of the temperature.^65^ Further evidence for the interaction of the unpaired electrons with the central aluminum nucleus in **3** was obtained from the pulsed^70^ electron spin echo envelop modulation (ESEEM) spectroscopy.^65^ As seen in Figure 4, the strong ESEEM pattern corresponds to the nuclear Larmor frequency (3.82 MHz) of the aluminum nucleus at X-band (9.7 GHz) with which the free electrons interact. DFT computations of the simplified **Λ-3-H** model (quartet state, UB3LYP/6-311G** level) reveal that while the unpaired electrons are largely localized on the C_2_S_2_ units of the three dithiolene ligands (the spin density of the C_2_S_2_ unit = 0.77), the central aluminum bears a spin density of −0.04.

Solid-state EPR spectra of **3** (Figure 3d) show similar general features to those observed for **3** in toluene (Figure 3b,c). At room temperature, the spectrum is an unresolved singlet with a line width of ca. 236 G (Figure S6).^65^ In the temperature range of 6–20 K, the spectrum remains an exchange-narrowed singlet at *g* = ca. 2.01 with a line width of 25 G (the line shape does not change, Figure S7).^65^ However, the spectrum starts to broaden above 20 K (Figure 3d). This broadening is almost perfectly Lorentzian with a width of ca. 15 G at 60 K (Figure S8),^65^ giving *T*_2_ < 4 ns at this temperature. The similar broadening observed at the same temperature interval for the powder and solution (in toluene) samples of **3** indicates that the short *T*_2_ relaxation time is not due to intermolecular interactions or crystal field effects but related to the triradical nature of **3**.

### SQUID Magnetometry

**Figure 5:**
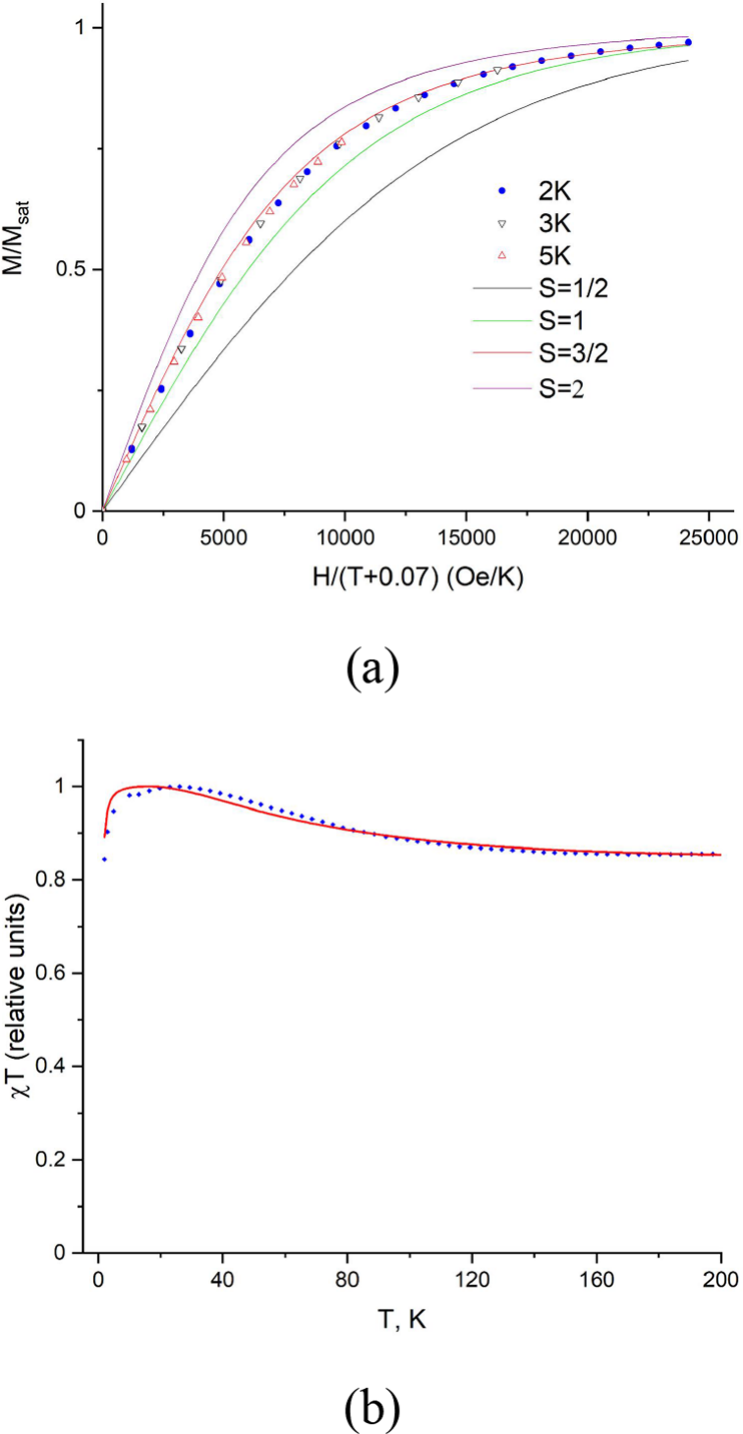
(a) *M/M*_sat_ vs *H/*(*T*–θ) plot, where θ = −0.07 K, at 2, 3, and 5 K. Solid lines correspond to Brillouin functions for *S* = 1/2, 1, 3/2, and 2. (b) Temperature dependence of the χ*T* product (blue dots) and its best fit to the formula χ*T* = *T*[*K*·*M*(*J*, *T*) + *B*] (see the Supporting Information) resulting in *J*/*k* = 30 K. The magnetic field is 10,000 Oe.

Magnetic data on the powder of **3** were collected using SQUID magnetometry. The magnetic moment versus magnetic field measurements were performed at 2, 3, and 5 K with the field range of −50000 to 50000 Oe, being swept twice in both directions and showing no evidence of the sample hysteresis. As seen in Figure 5a, the *M/M*_sat_ versus *H/*(*T*–θ) plot closely follows the Brillouin curve corresponding to *S* = 3/2, which unambiguously confirms the quartet ground state of triradical **3**.^21^

While the 1/χ versus *T* plot (Figure S9) approaches linearity (where χ is the magnetic susceptibility), the χ*T* product versus *T* plot (Figure 5b) allows for more detailed analyses of the doublet–quartet equilibrium.^21^ The drop at low temperatures (*T* = 2–5 K) is related to paramagnetic saturation. We also observe a decrease in magnetization with the temperature increase from 25 to 200 K. When *T* = 200 K, the χ*T* value is ca. 85% of its maximum at lower temperature. This decrease reveals an admixture of a thermally populated doublet excited state.^21^ In the presence of the quartet/doublet equilibrium, the net magnetization should be described by eq S1 (see the Supporting Information).^65^ As seen in Figure 5b, the experimental χ*T* versus *T* dependence can be reasonably well approximated by eq S1(65) with a doublet–quartet energy gap (Δ*E*_DQ_) of 3J/k (0.18 kcal mol^–1^, i.e., 90 K), which compares well to the theoretical value (Δ*E*_DQ_ = 0.14 kcal mol^–1^) of the **Λ-3-H** model obtained by the broken-symmetry DFT computations.^23,65^ Based on the Δ*E*_DQ_ value (0.18 kcal mol^–1^) of **3**, a substantial (more than 5% of the spins) population of the doublet should be presented above 30 K. At 298 K, the populations of the quartet and doublet states should be similar, given the Boltzmann factor of ca. 0.75. For the reported triradical species,^21,57^ simulations of distinct EPR spectra corresponding to the quartet and doublet states were used to analyze the experimental data. However, for **3,** the broadening of EPR lines due to the very short relaxation times (Figures 3b and S5) makes fine spectral features unresolved at temperatures with appreciable fraction of spins in the doublet state, thus preventing our study on the doublet–quartet equilibrium by EPR.

### Molecular Structure and DFT Computations

**Figure 6:**
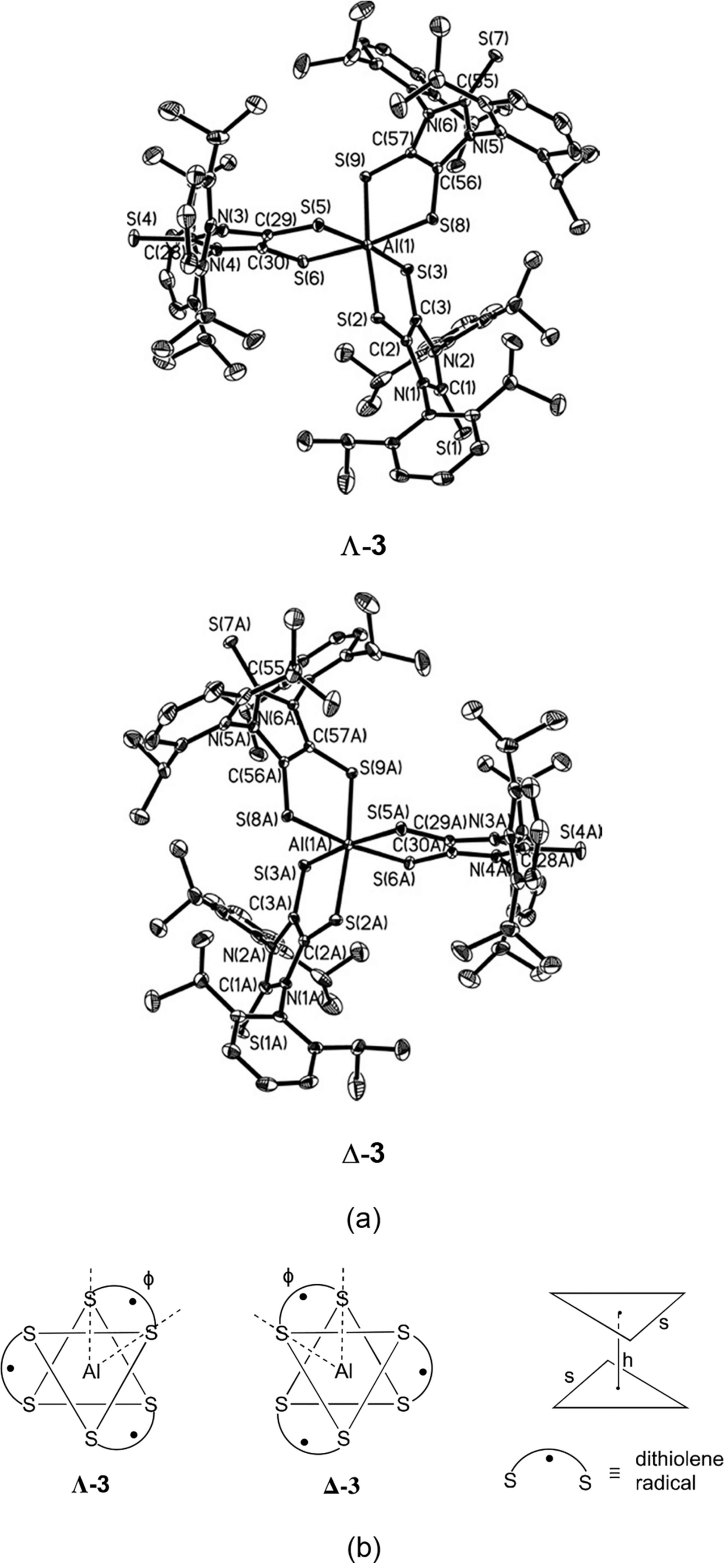
(a) Molecular structures of enantiomeric **3** (thermal ellipsoids represent 30% probability; hydrogen atoms on carbons are omitted for clarity). Selected bond distances (Å) and angles (°): C(1)–S(1) 1.6479(19), C(2)–C(3) 1.407(2), C(2)–S(2) 1.6859(18), C(3)–S(3) 1.6861(18), S(2)–Al(1) 2.4200(7), S(3)–Al(1) 2.4092(7); S(2)–C(2)–C(3) 126.40(13), C(2)–S(2)–Al(1) 98.03(6), S(2)–Al(1)–S(3) 90.33(2), and S(2)–Al(1)–S(9) 173.84(3). (b) Schematic representation of enantiomers of **3** (trigonal twist angle ϕ, triangle side *s*, and intertriangle distance *h*).

**Figure 7:**
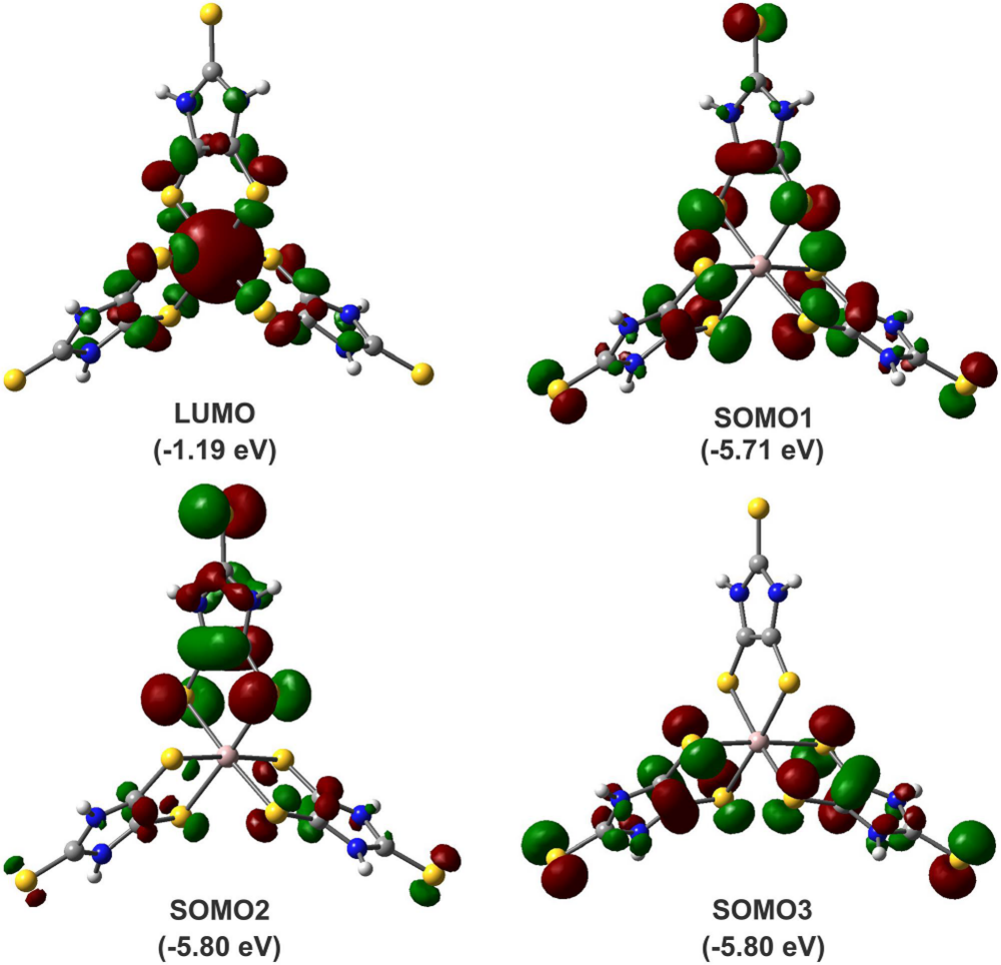
LUMO (*a*_1_), SOMO1 (*a*_2_), doubly degenerate SOMO2 (*e*) and SOMO3 (*e*) orbitals of the simplified **Λ-3-H** model in quartet ground state. Orbital energies are shown in parentheses.

X-ray structural analysis reveals that **3** exists as a pair of enantiomers with identical bonding parameters (Figure 6a). The central aluminum atom in **3** is coordinated by six sulfur atoms from three dithiolene ligands. The steric bulk of the dithiolene ligand (Figure S10) provides sufficient kinetic stability such that **3** can be isolated. The structural distortion from the regular octahedron for tris(bidentate ligand) complexes has been evaluated with both trigonal twist angle (ϕ) and *s*/*h* ratio (Figure 6b).^2,71,72^ The six-coordinate aluminum atom in **3** adopts an octahedral geometry with an elongated distortion [ϕ = 52.1° (av), *s*/*h* = 1.10 (av) vs ϕ = 60.0°, *s*/*h* = 1.22 for a regular octahedron^71^], which compares to that [ϕ = 55.1° (av), *s*/*h* = 1.16 (av)] of the quartet state **Λ-3-H** model. While being marginally shorter than those (2.470 Å, av) in **Λ-3-H**, the Al–S bonds in **3** [2.4092(7)–2.4307(7) Å] are somewhat longer than those [2.2785(10)–2.293(4) Å] for the four-coordinate aluminum-based ethene–tetrathiolate complex.^73^ The Al–S bonds in **Λ-3-H** [with a Wiberg bond index (WBI) value of 0.55] are evidently polarized (ca. 84% toward sulfur and ca. 16% toward aluminum). The C_2_S_2_Al rings in **3** are almost planar [bend angle (η) between the AlS_2_ plane and the S_2_C_2_ plane = 2.4° (av)], similar to that for **Λ-3-H** (0°). The olefinic C–C bonds [1.408(2) Å, av] and C–S bonds [1.6855(17) Å, av] in the dithiolene units of **3** compare well to those in **Λ-3-H** (*d*_C–C_ = 1.418 Å, *d*_C–S_ = 1.701 Å) and in monoanionic radical **2** [*d*_C–C_ = 1.417(3) Å, *d*_C–S_ = 1.677(3) Å, av].^60^ However, they are in contrast to those for the reported dithiolate complexes, which contain relatively shortened olefinic C–C bonds and elongated C–S bonds [i.e., for dianionic (C_3_S_5_)^2–^, *d*_C–C_ = 1.371(8) Å, *d*_C–S_ = 1.724(6) Å].^74^ The X-ray structural data support the monoanionic radical essence of the dithiolene ligands in **3**. Thus, the central aluminum atom in **3** is in the formal oxidation state of +3. Natural bond orbital (NBO) analysis shows that while the aluminum atom in **Λ-3-H** has a positive natural charge of +0.57, each sulfur atom next to the central aluminum has a negative natural charge of −0.04. Our computational studies of the **Λ-3-H** model in the quartet ground state (Figure 7) show that LUMO (*a*_1_) mainly involves the aluminum–sulfur σ-antibonding interaction, whereas SOMO1 (*a*_2_) and doubly degenerate SOMO2 (*e*) and SOMO3 (*e*) orbitals are virtually ligand-based, including C–C π-bonding and C–S π-antibonding features.

## Conclusion

Compound **3**, the first tris(dithiolene) triradical, has been prepared via a low-temperature reaction of **2** with aluminum iodide. The low-temperature EPR spectroscopic studies of **3** show that the temperature dependence of the intensity of the Δ*m* = 2 and Δ*m* = 3 transitions follows the Curie law, supporting the quartet ground state triradical essence of **3**. The quartet ground state triradical character of **3** was further confirmed by SQUID magnetometry. Both SQUID (Δ*E*_DQ_ = 0.18 kcal mol^–1^) and broken-symmetry DFT computations (Δ*E*_DQ_ = 0.14 kcal mol^–1^) reveal a small doublet–quartet energy gap for **3**.

## Supporting Information Available

Syntheses, spectral data, computations, and X-ray crystal determination (PDF)
Cartesian coordinates for the **Λ-3-H** models (quartet state and broken-symmetry doublet state) (XYZ)

## Supplementary Material

ja4c05631_si_001.pdf

ja4c05631_si_002.xyz
