# Supplementary material for: A Stable Aluminum Tris(dithiolene) Triradical
Source: J Am Chem Soc. 2024 May 31;146(23):16340–7. doi: 10.1021/jacs.4c05631 (PMC11177253; doi:10.1021/jacs.4c05631)
Supplement: Supplementary file 1 — ja4c05631_si_001.pdf [file ja4c05631_si_001.pdf]

SUPPORTING INFORMATION

A Stable Aluminum Tris(dithiolene) Triradical

Phuong M. Tran,† Yuzhong Wang,† Boris Dzikovski,‡ Mitchell E. Lahm,† Yaoming Xie,† Pingrong Wei,† Vladislav V. Klepov,† Henry F. Schaefer III† and Gregory H. Robinson†*

† Department of Chemistry and the Center for Computational Chemistry, The University of Georgia, Athens, Georgia 30602-2556 (USA)

‡ Department of Chemistry and Chemical Biology, and ACERT, National Biomedical Center for Advanced Electron Spin Resonance Technology, Cornell University, Ithaca, New York 14853-1301 (USA)

To whom correspondence should be addressed. Email: robinson@uga.edu

Table of Contents

1. **Synthesis of 3** **S3**
2. **EPR study of 3** **S4-S11**
3. **SQUID study of 3** **S12-S15**
4. **DFT computations of the simplified Λ-3-H model** **S17-S20**
5. **X-ray structural analysis of 3** **S16, S21-S27**
6. **References** **S27**

SUPPORTING INFORMATION of SYNTHESES

Materials and Methods

General.

The syntheses of air-sensitive compounds were performed under purified argon using Schlenk techniques and an inert atmosphere drybox (M-Braun LabMaster SP). Chemicals were purchased from Aldrich and Strem and used as received. The solvents were dried and distilled under argon from Na/benzophenone prior to use. EPR measurements were done in ACERT. CW EPR spectra were recorded on a Bruker ElexSys E500 EPR spectrometer at 9.4 GHz. For low temperature measurements an ESR910 liquid-helium cryostat (Oxford Instruments) was used. ESEEM and relaxation times measurements were performed on a Bruker ElexSys E580 EPR spectrometer at 9.7 GHz supplied with a FlexLine Systems cryogen-free cryostat. EasySpin software package (5.2.35 version) was used for simulations of EPR spectra.[1] X-ray intensity data for **(3)$_2$·(toluene)$_3$** were measured at 135K on a Bruker D8 Quest PHOTON 100 CMOS X-ray diffractometer system with Incoatec Microfocus Source (IμS) monochromated Mo Kα radiation (λ = 0.71073 Å, sealed tube) using phi and omega-scan technique. The UV-visible absorption spectrum of **3** was recorded under argon gas protection in 1 cm cuvettes using a Varian Cary 5000 UV-vis-NIR spectrophotometer. SQUID measurements were carried out using a Quantum Design MPMS-3 magnetometer in the Cornell Center for Materials Research.

Compound **3**: 150 mL of hexane was added to an aluminum foil wrapped 250 mL Schlenk flask containing both **2** (1.000 g, 1.58 mmol) and aluminum iodide (0.214 g, 0.525 mmol) at -78 ºC. The mixture was allowed to gradually warm to the room temperature overnight and stirred for an additional 4 h. After filtration, the volatile materials were removed from the filtrate in vacuo. The resulting residue was recrystallized in toluene/hexane mixed solvent at -40 ºC, giving **3** as X-ray quality dark blue crystals (0.142 g, 16.7% yield). Mp: gradually decomposed (> 115.3 ºC) and melt (>275.8 ºC). UV-vis (λ/nm): 423, 435, 595 and 645. Crystal data for **(3)$_2$·(toluene)$_3$**: $C_{183}H_{228}Al_2N_{12}S_{18}$, fw = 3226.80, monoclinic, C2/c, a = 16.2816(19) Å, b = 26.410(3) Å, c = 43.555(5) Å, β = 96.921(3)°, V = 18592(4) Å^3, Z = 4, R1 = 0.0435 for 17621 data ($I > 2\sigma(I)$), wR_2 = 0.1091 (all data).

SUPPORTING INFORMATION of EPR MEASUREMENT

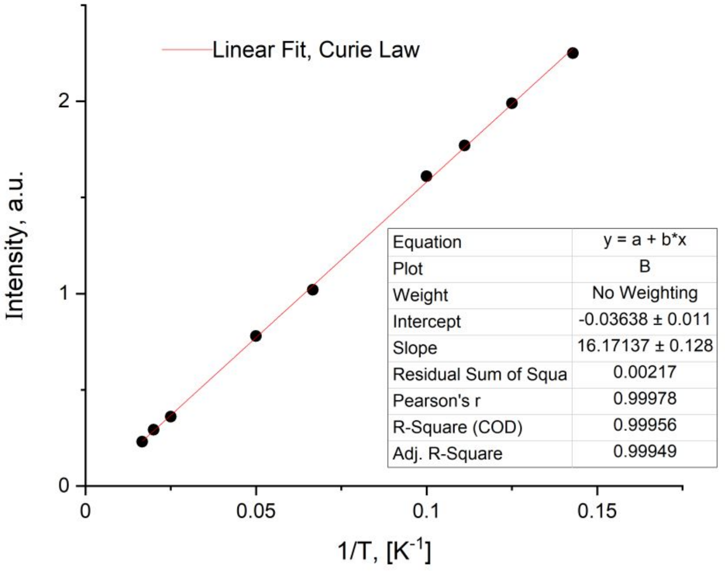

(a)

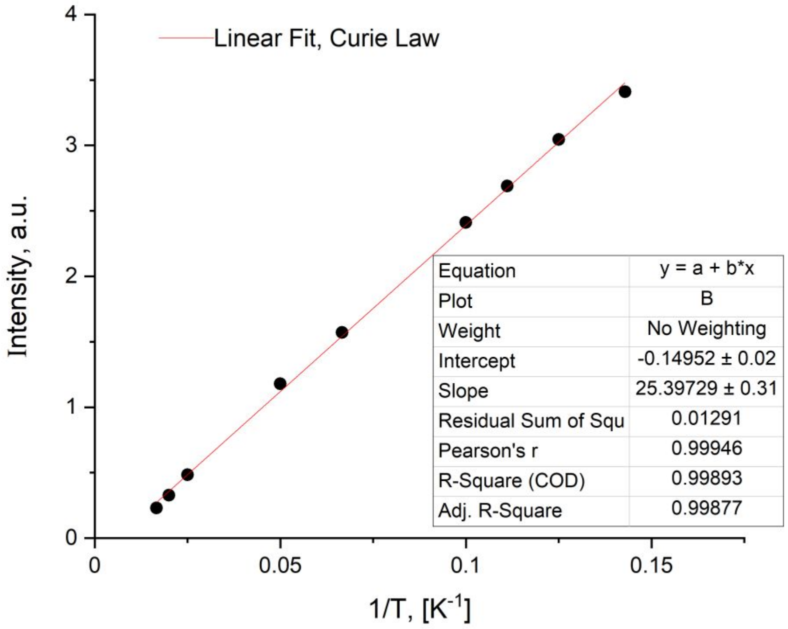

(b)

Figure S1. Temperature dependence of EPR intensity of the Δm = 2 (a) and Δm= 3 (b) transitions of **3** (7–60 K).

Simulation of the broad feature of the main spectral line ($\Delta m = 1$) using the value of dipolar splitting estimated from the intensity of forbidden transitions

The main ($\Delta m = 1$) line of the EPR spectrum consists of a relatively sharp central peak and broad features which spread about 1500 G. The features are more pronounced in the absorption form (the first integral of the initial cw spectrum). Figure S2 shows an EasySpin simulation using the dipolar splitting estimate obtained from the intensity of forbidden transitions (355 G) and a very large value of D strain. The simulation adequately depicts the relative intensities of the broad and narrow features.

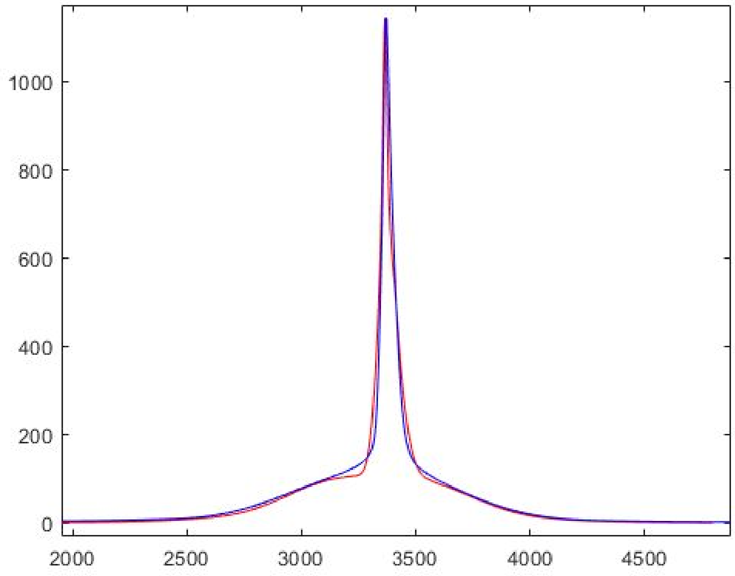

Figure S2. The first integral of the cw EPR spectrum (blue) of **3** recorded at 10 K and its EasySpin simulation (red) using D=355 G and DStrain=1200 MHz.

Monoradical impurities observed in the room-temperature EPR spectrum of 3

The sharp line in Figure 3a corresponds to a monoradical admixture, which amounts to only a small fraction (< 0.5%) of the total amount of spins. The monoradical may be produced due to the partial decomposition of **3**, which is extremely sensitive to oxygen and moisture. As seen in Figure S3, the line has some partially resolved hyperfine structure, which can be best simulated with a A_N value of 1.4 G. It is almost the same as that for the reported naked dithiolene radical anion.[2] There is no sign of interaction between the free electron and aluminum nuclei. Incomplete resolution of the hyperfine lines is likely due to the broadening from Heisenberg exchange with high concentration of triradical in the solution.

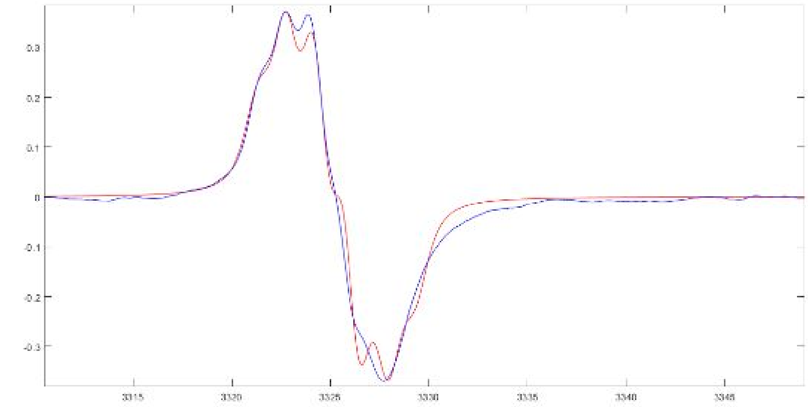

Figure S3. A zoom of the intense narrow line from Figure 3a (blue) and its EasySpin simulation (red). The hyperfine feature corresponds to a splitting of 1.4 G. The line asymmetry may be due to the effects of slow motion.

Spin-lattice and spin-spin relaxation times of aluminum dithiolene triradical in frozen toluene matrix

The spin-lattice relaxation time T_1 was measured at 10 and 15K using the picket-and-fence saturation recovery technique[3] designed to exclude spin diffusion effects (Figure S4).

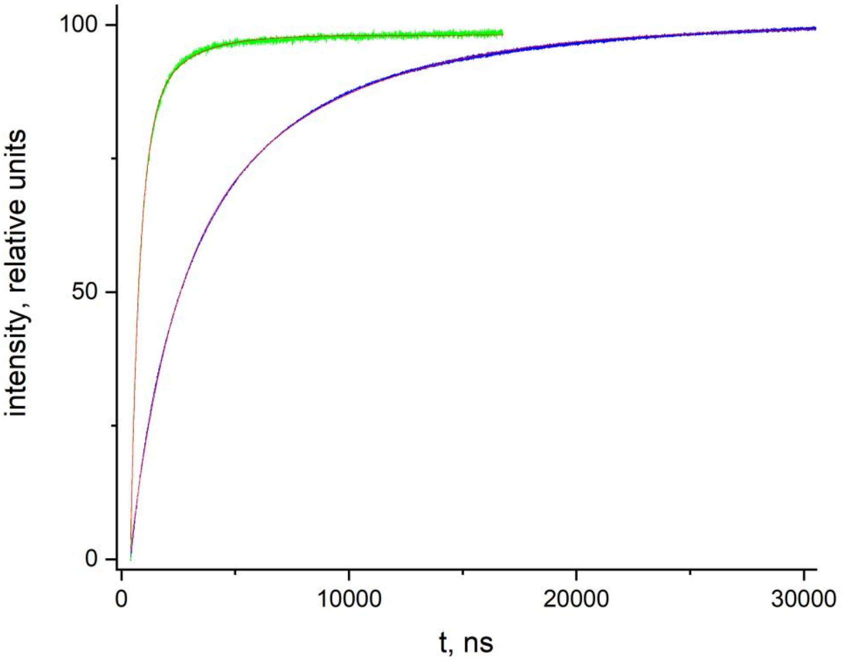

Figure S4. Saturation recovery curves obtained at 10 K (blue) and 15 K (green) and their fitting with a biexponential expression: $I = 100–A_1exp(t/\tau_1)–A_2exp(t/\tau_2)$. At 10 K, A_1 = 54.0, τ_1 = 1713 ns, A_2 = 46.0, τ_2 = 6966 ns; at 15 K, A_1 = 90.6, τ_1 = 412 ns, A_2 = 9.4, τ_2 = 1812 ns.

Although the explanation for the biexponential behavior in Figure S4 is not quite clear, the figure shows that the spin-lattice relaxation time for **3** is unusually short for the low temperature, which is less than a microsecond at 15K and single microseconds at 10 K. For comparison, at 10–20 K for most organic radicals and paramagnetic metal compounds (Cu, Mn, Cr), the common values of spin-lattice relaxation times are in a range of dozens of microseconds up to milliseconds.[4] This short relaxation time is likely related to the triradical nature of **3**.

A rough estimate of the spin-spin relaxation time can be made using electron spin echo decay curves. As seen in Figure S5 the ESE decay curves are strongly affected by the ESEEM oscillations. It makes the exact determination of T_2 difficult, but approximate values of this parameter can be obtained by exponential fitting of the whole curve or its part. Although the fits shown in Figure S5 do not exactly follow the ESE curves, they seem to adequately model the rate of decay and give approximate values for T_2 (ca. 650 ns at 10 K and ca. 290 ns at 15 K). The echo signal practically disappears at ca. 25 K,

which points to further shortening of T_2 time below 50 ns. This observation is consistent with the detectable broadening of cw EPR spectra starting at approximately this temperature. T_2 of 50 ns would correspond to a Lorentzian broadening of $1/\gamma T_2$ = 1.14 G, which can be detectable in a cw spectrum.

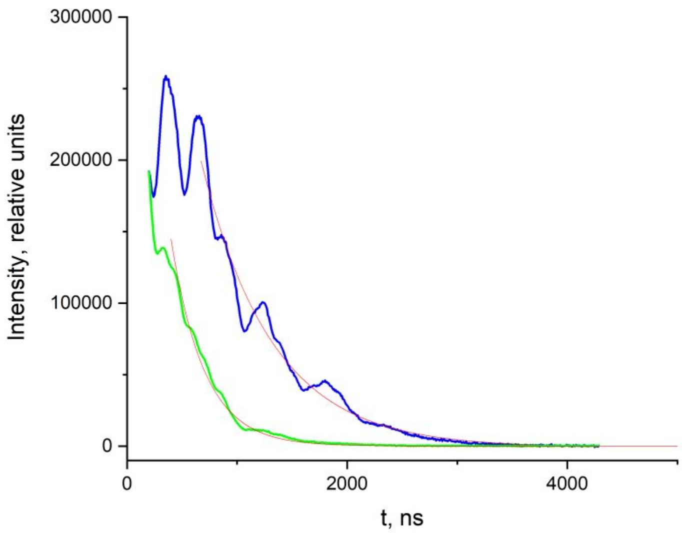

Figure S5. ESE decay curves at 10 K (blue) and 15 K (green). The approximate estimate of T_2 relaxation times is ca. 650 and 290 ns respectively.

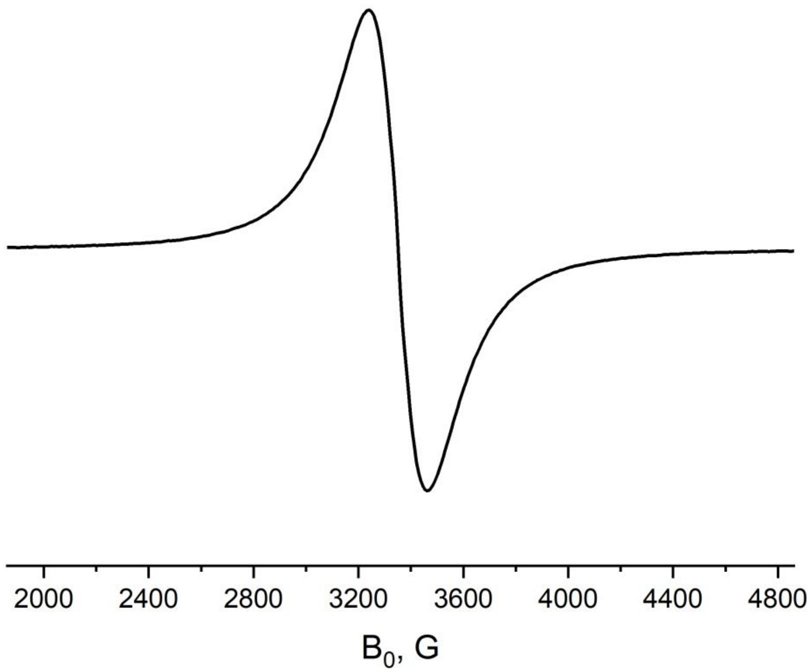

Figure S6. Solid-state X-band EPR spectrum of **3** (at room temperature).

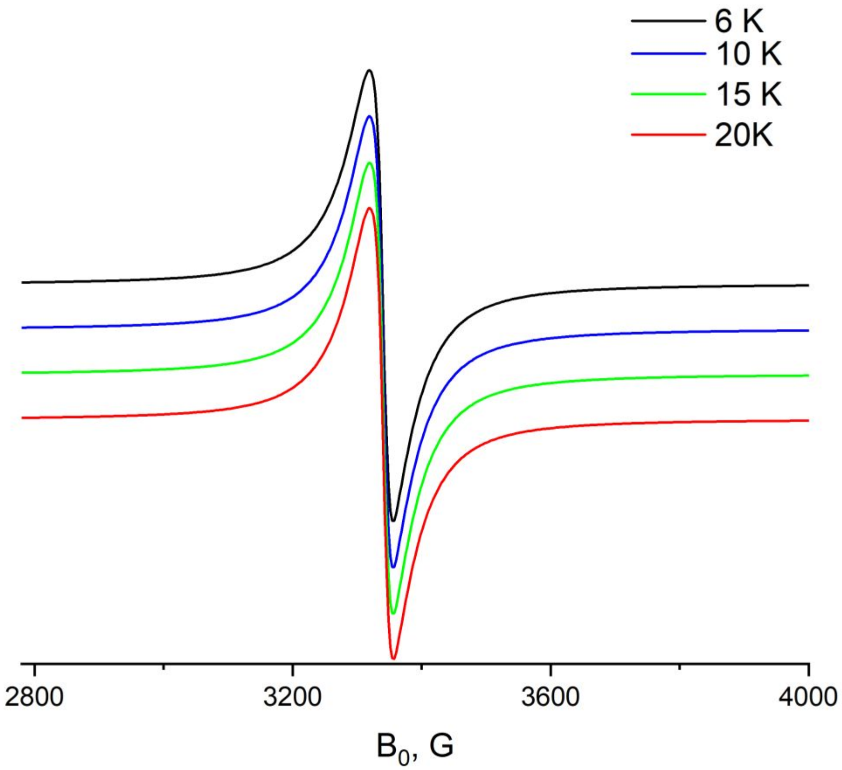

Figure S7. Solid-state X-band EPR spectra of **3** at 6–20 K.

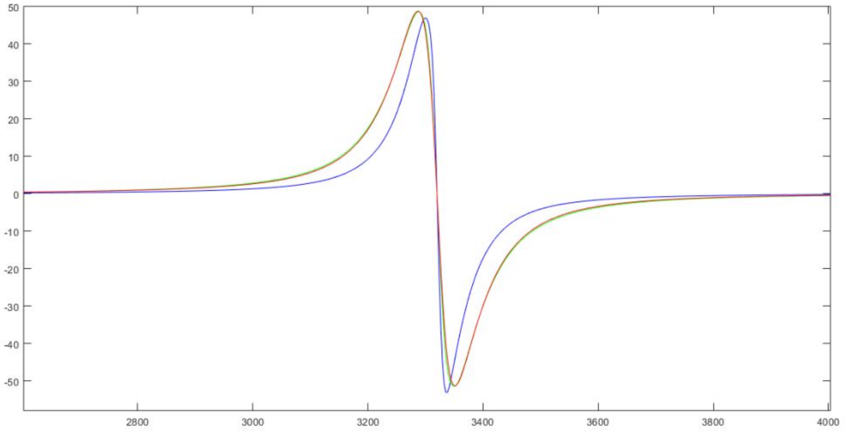

Figure S8. Simulation of the EPR line shape of solid **3** at 60 K by convolution of the 20 K spectrum and a Lorentzian line with a width of 15 G.

The sample preparation for SQUID experiments

The sample holder, a 3.5 mm OD quartz tube, was pulled from a standard 4 mm OD EPR tube, flame-sealed at one end and cut to a length of approximately 10 mm. It was then filled almost to the middle by argon saturated eicosane and transferred to the anaerobic chamber where approximately 15 mg of the aluminum dithiolene radical powder was added. The part of the holder above the sample was tightly packed with solid eicosane powder to immobilize the triradical powder and sealed with epoxy to prevent oxygen access.

Analytical expression for the net magnetization in the presence of thermal quartet – doublet equilibrium

Our spin system can be approximated by an equilateral triangle of three spins S_1, S_2 and S_3 with a Heisenberg Hamiltonian of

$$H = -J[\vec{S}^2 - \vec{S}_1^2 - \vec{S}_2^2 - \vec{S}_3^2]$$

Where J is the spin-coupling constant.

The eigenvalues are given by E = – $JS(S+1) + 3JS_e(S_e + 1)$, where $S_1 = S_2 = S_3 = S_e = 1/2$ and S (total spin) = 1/2 (2-fold degenerate with no magnetic field), 3/2 (4-fold degenerate)

In the presence of magnetic field H, we also have Zeeman splitting:

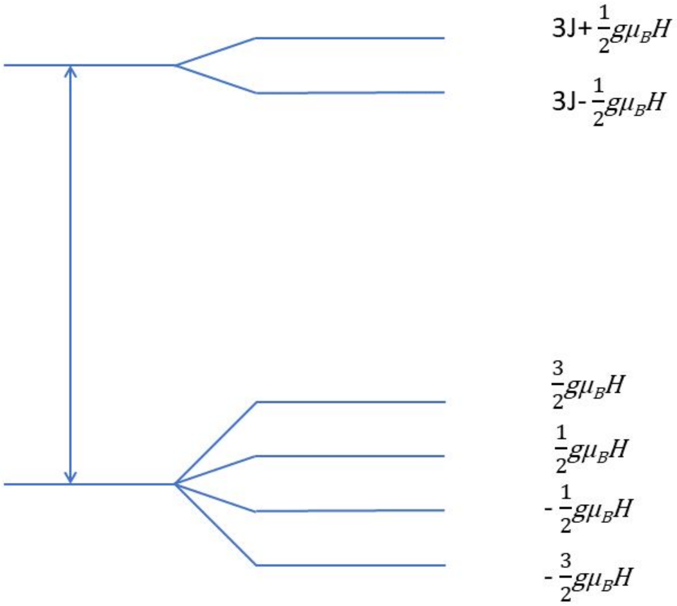

The temperature dependence of the net magnetization for our three-spin system can be calculated by a procedure similar to one described for a biradical.[5]

We start with a partition function:

$$Z = \sum \exp\left(-\frac{E_i}{kT}\right)$$

$$Z = \exp(-\tfrac{3}{2}g\mu_B H/kT) + \exp(-\tfrac{1}{2}g\mu_B H/kT) + \exp(\tfrac{1}{2}g\mu_B H/kT) + \exp(\tfrac{3}{2}g\mu_B H/kT)$$
$$+ \exp(-\tfrac{3J}{kT})\left(\exp\left(-\tfrac{1}{2}g\mu_B H/kT\right) + \exp(\tfrac{1}{2}g\mu_B H/kT)\right)$$
$$= 2[\cosh(\tfrac{3}{2}g\mu_B H/kT) + \cosh(\tfrac{1}{2}g\mu_B H/kT) + \exp(-\tfrac{3J}{kT})\cosh(\tfrac{1}{2}g\mu_B H/kT)]$$

Then the magnetization can be estimated as $M = NkT(\frac{\delta \ln(Z)}{\delta H})_T$

Which gives:

$$Ng\mu_B[3\sinh(\tfrac{3}{2}g\mu_B H/kT) + \sinh(\tfrac{1}{2}g\mu_B H/kT) + \exp(-\tfrac{3J}{kT})\sinh(\tfrac{1}{2}g\mu_B H/kT)]/Z$$

or

$$M = Ng\mu_B[3\sinh(\tfrac{3}{2}AH/T) + \sinh(\tfrac{1}{2}AH/T) + \exp(-\tfrac{3J}{kT})\sinh(\tfrac{1}{2}AH/T)]/Z \qquad \text{eq. S1}$$

$$Z = 2[\cosh(\tfrac{3}{2}AH/T) + \cosh(\tfrac{1}{2}AH/T) + \exp(-\tfrac{3J}{kT})\cosh(\tfrac{1}{2}AH/T)]$$

Where $A = \frac{1}{2}g\mu_B/k$ = 1.344 x 10^{-4} K/Gauss

Our experimental data for χT vs T were fitted to the expression:

$\chi T = T\{K \cdot M(J, T) + B\}$

Where K is a fudge factor accounting for the total number of spins in the sample and B is a temperature-independent correction for a non-paramagnetic admixture as in reference [6].[6]

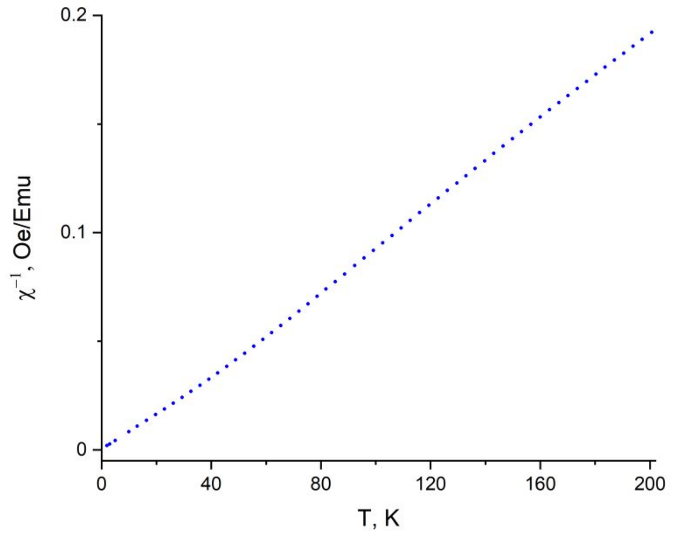

Figure S9. Inverse susceptibility vs. temperature plot for **3**.

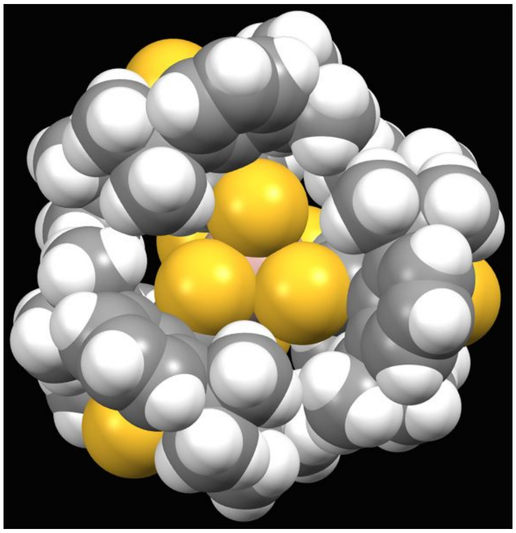

Figure S10. Space filling model of **Λ-3** (yellow, sulfur; pink, aluminum; grey, carbon; white, hydrogen).

SUPPORTING INFORMATION of COMPUTATIONS

All computations employed the Gaussian 16 (Revision C.01) program:

Gaussian 16, Revision C.01,
M. J. Frisch, G. W. Trucks, H. B. Schlegel, G. E. Scuseria,
M. A. Robb, J. R. Cheeseman, G. Scalmani, V. Barone,
G. A. Petersson, H. Nakatsuji, X. Li, M. Caricato, A. V. Marenich,
J. Bloino, B. G. Janesko, R. Gomperts, B. Mennucci, H. P. Hratchian,
J. V. Ortiz, A. F. Izmaylov, J. L. Sonnenberg, D. Williams-Young,
F. Ding, F. Lipparini, F. Egidi, J. Goings, B. Peng, A. Petrone,
T. Henderson, D. Ranasinghe, V. G. Zakrzewski, J. Gao, N. Rega,
G. Zheng, W. Liang, M. Hada, M. Ehara, K. Toyota, R. Fukuda,
J. Hasegawa, M. Ishida, T. Nakajima, Y. Honda, O. Kitao, H. Nakai,
T. Vreven, K. Throssell, J. A. Montgomery, Jr., J. E. Peralta,
F. Ogliaro, M. J. Bearpark, J. J. Heyd, E. N. Brothers, K. N. Kudin,
V. N. Staroverov, T. A. Keith, R. Kobayashi, J. Normand,
K. Raghavachari, A. P. Rendell, J. C. Burant, S. S. Iyengar,
J. Tomasi, M. Cossi, J. M. Millam, M. Klene, C. Adamo, R. Cammi,
J. W. Ochterski, R. L. Martin, K. Morokuma, O. Farkas,
J. B. Foresman, and D. J. Fox, Gaussian, Inc., Wallingford CT, 2019.

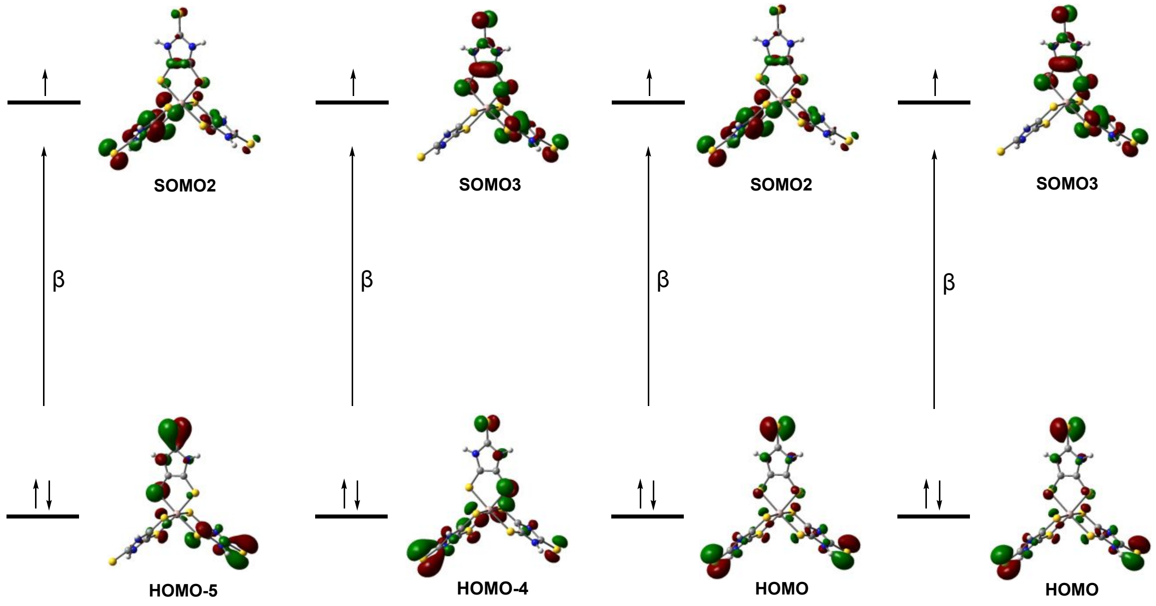

Figure S11. Electronic transitions that contribute predominantly to the absorption bands (545 and 594 nm) in the UV-vis spectrum of the **Λ-3-H** model calculated from TD-DFT computations (UB3LYP/6-311G**, SMD, toluene). While the absorption at 545 nm mainly involves the HOMO-5 $\rightarrow$ SOMO2 and HOMO-4 $\rightarrow$ SOMO3 excitations, the absorption at 594 nm mainly involves the HOMO $\rightarrow$ SOMO2 and HOMO $\rightarrow$ SOMO3 excitations.

Table S1. Coordinates of the UB3LYP/6-311G** optimized geometry of the **Λ-3-H** model, quartet state (in D_3 symmetry).

Center Number	Atomic Number	Atomic Type	Coordinates (Angstroms) X	Y	Z
1	13	0	0.000000	0.000000	0.000000
2	16	0	0.000000	6.941971	0.000000
3	16	0	0.916011	1.755773	1.475406
4	16	0	-0.916011	1.755773	-1.475406
5	16	0	6.011923	-3.470985	0.000000
6	16	0	1.062539	-1.671175	1.475406
7	16	0	1.978549	-0.084598	-1.475406
8	16	0	-6.011923	-3.470985	0.000000
9	16	0	-1.978549	-0.084598	1.475406
10	16	0	-1.062539	-1.671175	-1.475406
11	7	0	0.573191	4.432972	0.919651
12	7	0	-0.573191	4.432972	-0.919651
13	7	0	3.552471	-2.712884	0.919651
14	7	0	4.125662	-1.720088	-0.919651
15	7	0	-4.125662	-1.720088	0.919651
16	7	0	-3.552471	-2.712884	-0.919651
17	6	0	0.000000	5.283838	0.000000
18	6	0	0.374282	3.111008	0.602152
19	6	0	-0.374282	3.111008	-0.602152
20	6	0	4.575938	-2.641919	0.000000
21	6	0	2.507071	-1.879642	0.602152
22	6	0	2.881353	-1.231367	-0.602152
23	6	0	-4.575938	-2.641919	0.000000
24	6	0	-2.881353	-1.231367	0.602152
25	6	0	-2.507071	-1.879642	-0.602152
26	1	0	4.671297	-1.456470	-1.726342
27	1	0	3.596989	-3.317227	1.726342
28	1	0	-4.671297	-1.456470	1.726342
29	1	0	-3.596989	-3.317227	-1.726342
30	1	0	1.074309	4.773697	1.726342
31	1	0	-1.074309	4.773697	-1.726342

Table S2. Coordinates of the UB3LYP/6-311G** optimized geometry of the **Λ-3-H** model, broken-symmetry doublet state (in C_2 symmetry).

Center Number	Atomic Number	Atomic Type	Coordinates (Angstroms) X	Y	Z
1	13	0	0.000000	0.000000	-0.002574
2	16	0	0.000000	0.000000	6.942385
3	16	0	1.474951	0.914504	1.755589
4	16	0	-1.474951	-0.914504	1.755589
5	16	0	0.000000	6.019776	-3.463271
6	16	0	1.474346	1.066148	-1.673790
7	16	0	-1.478156	1.975930	-0.090409
8	16	0	0.000000	-6.019776	-3.463271
9	16	0	1.478156	-1.975930	-0.090409
10	16	0	-1.474346	-1.066148	-1.673790
11	7	0	0.918126	0.575685	4.433347
12	7	0	-0.918126	-0.575685	4.433347
13	7	0	0.918590	3.557866	-2.711812
14	7	0	-0.920967	4.128827	-1.718090
15	7	0	0.920967	-4.128827	-1.718090
16	7	0	-0.918590	-3.557866	-2.711812
17	6	0	0.000000	0.000000	5.284208
18	6	0	0.601556	0.375006	3.111451
19	6	0	-0.601556	-0.375006	3.111451
20	6	0	-0.000709	4.581501	-2.638158
21	6	0	0.600250	2.510165	-1.881856
22	6	0	-0.604597	2.882518	-1.233779
23	6	0	0.000709	-4.581501	-2.638158
24	6	0	0.604597	-2.882518	-1.233779
25	6	0	-0.600250	-2.510165	-1.881856
26	1	0	-1.728180	4.673490	-1.454067
27	1	0	1.726044	3.604271	-3.314996
28	1	0	1.728180	-4.673490	-1.454067
29	1	0	-1.726044	-3.604271	-3.314996
30	1	0	1.724588	1.077164	4.774084
31	1	0	-1.724588	-1.077164	4.774084

The doublet-quartet energy gap (ΔE_{DQ}) of triradical **3** was calculated using the following equation (with the correction for spin contamination):[7]

$\Delta E_{DQ} = \Delta E_U[(\langle S^2_Q \rangle - 0.75)/(\langle S^2_Q \rangle - \langle S^2_{BS} \rangle)]$

For **3**,

$\Delta E_U = 0.09$ kcal.mol^{-1}

$\langle S^2_Q \rangle = 3.77$

$\langle S^2_{BS} \rangle = 1.76$

Thus, $\Delta E_{DQ} = 0.14$ kcal.mol^{-1}

SUPPORTING INFORMATION of X-RAY

Compound $(3)_2$·$(toluene)_3$

Table S3. Sample and crystal data for **$(3)_2$·$(toluene)_3$**.

**Identification code**	**$(3)_2$·$(toluene)_3$**	
**Chemical formula**	$C_{183}H_{228}Al_2N_{12}S_{18}$	
**Formula weight**	3226.80 g/mol	
**Temperature**	135(2) K	
**Wavelength**	0.71073 Å	
**Crystal size**	0.140 x 0.240 x 0.280 mm	
**Crystal system**	monoclinic	
**Space group**	C2/c (No. 15)	
**Unit cell dimensions**	a = 16.2816(19) Å	α = 90°
	b = 26.410(3) Å	β = 96.921(3)°
	c = 43.555(5) Å	γ = 90°
**Volume**	18592(4) Å^3	
**Z**	4	
**Density (calculated)**	1.153 g/cm^3	
**Absorption coefficient**	0.269 mm^{-1}	
**F(000)**	6896	

Table S4. Data collection and structure refinement for **$(3)_2\cdot$(toluene)$_3$**.

**Theta range for data collection**	1.94 to 27.48°
**Index ranges**	-21<=h<=21, -34<=k<=34, -56<=l<=56
**Reflections collected**	252582
**Independent reflections**	21300 [R(int) = 0.0467]
**Max. and min. transmission**	0.7456 and 0.7007
**Structure solution technique**	direct methods
**Structure solution program**	SHELXT 2014/5 (Sheldrick, 2014)
**Refinement method**	Full-matrix least-squares on F^2
**Refinement program**	SHELXL-2018/3 (Sheldrick, 2018)
**Function minimized**	Σ w(F_o^2 - F_c^2)^2
**Data / restraints / parameters**	21300 / 190 / 1010
**Goodness-of-fit on F^2**	1.040
**Δ/σ_{max}**	0.003
**Final R indices**	17621 data; I>2σ(I) R1 = 0.0435, wR2 = 0.1024
	all data R1 = 0.0571, wR2 = 0.1091
**Weighting scheme**	w=1/[$\sigma^2(F_o^2)+(0.0442P)^2+27.9552P$]<br>where $P=(F_o^2+2F_c^2)/3$
**Largest diff. peak and hole**	0.959 and -0.475 eÅ^{-3}
**R.M.S. deviation from mean**	0.050 eÅ^{-3}

Table S5. Bond lengths (Å) for **(3)$_2$·(toluene)$_3$**.

Al1-S3	2.4092(7)	Al1-S9	2.4103(7)
Al1-S5	2.4092(7)	Al1-S6	2.4186(7)
Al1-S2	2.4200(7)	Al1-S8	2.4307(7)
S1-C1	1.6479(19)	S2-C2	1.6859(18)
S3-C3	1.6861(18)	S4-C28	1.6463(18)
S5-C29	1.6847(17)	S6-C30	1.6846(17)
S7-C55	1.6453(17)	S8-C56	1.6856(17)
S9-C57	1.6862(17)	N1-C2	1.376(2)
N1-C1	1.382(2)	N1-C16	1.443(2)
N2-C3	1.373(2)	N2-C4	1.444(2)
N2-C1	1.383(2)	N3-C29	1.377(2)
N3-C28	1.378(2)	N3-C43	1.443(2)
N4-C30	1.373(2)	N4-C28	1.381(2)
N4-C31	1.440(2)	N5-C55	1.384(2)
N5-C56	1.376(2)	N5-C70	1.444(2)
N6-C57	1.372(2)	N6-C55	1.379(2)
N6-C58	1.440(2)	C2-C3	1.407(2)
C4-C5	1.396(3)	C4-C9	1.392(4)
C5-C6	1.399(4)	C5-C13'	1.515(11)
C5-C13	1.515(12)	C6-C7	1.369(5)
C7-C8	1.370(5)	C8-C9	1.398(3)
C9-C10'	1.549(10)	C9-C10	1.488(9)
C10-C12	1.528(10)	C10-C11	1.527(9)
C10'-C12'	1.519(11)	C10'-C11'	1.540(12)
C13-C15	1.554(12)	C13-C14	1.538(12)
C13'-C15'	1.563(12)	C13'-C14'	1.540(11)
C16-C21	1.392(3)	C16-C17	1.394(3)
C17-C18	1.397(3)	C17-C25	1.516(3)
C18-C19	1.374(3)	C19-C20	1.376(3)
C20-C21	1.397(3)	C21-C22	1.511(3)
C22-C23	1.515(3)	C22-C24	1.521(3)
C25-C26	1.523(3)	C25-C27	1.527(4)
C29-C30	1.409(2)	C31-C36	1.391(3)
C31-C32	1.389(3)	C32-C33	1.398(3)
C32-C40	1.514(3)	C33-C34	1.367(4)
C34-C35	1.372(4)	C35-C36	1.387(3)
C36-C37	1.512(3)	C37-C38	1.522(4)
C37-C39	1.522(4)	C40-C41	1.524(4)
C40-C42	1.534(4)	C43-C48	1.395(3)
C43-C44	1.393(3)	C44-C45	1.394(3)

C44-C52	1.509(3)	C45-C46	1.372(4)
C46-C47	1.379(3)	C47-C48	1.393(3)
C48-C49	1.520(3)	C49-C51	1.528(4)
C49-C50	1.516(3)	C52-C53	1.515(3)
C52-C54	1.520(4)	C56-C57	1.407(2)
C58-C63	1.391(3)	C58-C59	1.393(3)
C59-C60	1.396(3)	C59-C67	1.510(3)
C60-C61	1.375(3)	C61-C62	1.371(3)
C62-C63	1.391(3)	C63-C64	1.517(3)
C64-C66	1.520(4)	C64-C65	1.533(4)
C67-C69	1.524(3)	C67-C68	1.526(3)
C70-C75	1.395(3)	C70-C71	1.389(3)
C71-C72	1.401(3)	C71-C79	1.510(3)
C72-C73	1.374(4)	C73-C74	1.374(4)
C74-C75	1.396(3)	C75-C76	1.521(3)
C76-C77	1.521(3)	C76-C78	1.523(3)
C79-C80	1.520(3)	C79-C81	1.531(3)
C82-C88	1.485(4)	C82-C83	1.404(4)
C82-C87	1.389(4)	C83-C84	1.363(4)
C84-C85	1.375(4)	C85-C86	1.345(4)
C86-C87	1.355(4)	C89-C90	1.39
C89-C94	1.39	C89-C95	1.564(9)
C90-C91	1.39	C91-C92	1.39
C92-C93	1.39	C93-C94	1.39

Table S6. Bond angles (°) for **$(3)_2$·(toluene)$_3$**.

S3-Al1-S9	84.85(2)	S3-Al1-S5	173.83(3)
S9-Al1-S5	98.36(2)	S3-Al1-S6	84.49(2)
S9-Al1-S6	84.49(2)	S5-Al1-S6	90.56(2)
S3-Al1-S2	90.33(2)	S9-Al1-S2	173.84(3)
S5-Al1-S2	86.80(2)	S6-Al1-S2	98.88(2)
S3-Al1-S8	99.07(2)	S9-Al1-S8	90.04(2)
S5-Al1-S8	86.24(2)	S6-Al1-S8	173.20(3)
S2-Al1-S8	86.94(2)	C2-S2-Al1	98.03(6)
C3-S3-Al1	98.02(6)	C29-S5-Al1	97.87(6)
C30-S6-Al1	97.76(6)	C56-S8-Al1	98.06(6)
C57-S9-Al1	98.19(6)	C2-N1-C1	110.87(14)
C2-N1-C16	124.98(15)	C1-N1-C16	123.13(14)
C3-N2-C4	123.22(15)	C3-N2-C1	110.80(15)
C4-N2-C1	125.98(15)	C29-N3-C28	111.08(14)
C29-N3-C43	124.51(14)	C28-N3-C43	123.45(14)
C30-N4-C28	111.02(14)	C30-N4-C31	124.33(14)
C28-N4-C31	124.64(14)	C55-N5-C56	110.81(14)
C55-N5-C70	122.85(14)	C56-N5-C70	125.39(14)
C57-N6-C55	111.00(13)	C57-N6-C58	124.16(14)
C55-N6-C58	124.83(14)	N1-C1-N2	105.04(15)
N1-C1-S1	127.39(14)	N2-C1-S1	127.57(14)
N1-C2-C3	106.49(15)	N1-C2-S2	127.07(13)
C3-C2-S2	126.40(13)	N2-C3-C2	106.76(15)
N2-C3-S3	126.28(14)	C2-C3-S3	126.88(14)
N2-C4-C5	117.3(2)	N2-C4-C9	117.8(2)
C5-C4-C9	124.8(2)	C6-C5-C4	115.7(3)
C6-C5-C13'	123.4(9)	C4-C5-C13'	120.7(9)
C6-C5-C13	120.7(11)	C4-C5-C13	123.5(10)
C7-C6-C5	121.3(3)	C8-C7-C6	120.9(3)
C7-C8-C9	121.3(3)	C8-C9-C4	115.9(3)
C8-C9-C10'	124.7(7)	C4-C9-C10'	119.4(7)
C8-C9-C10	119.5(6)	C4-C9-C10	124.6(6)
C12-C10-C11	109.6(8)	C12-C10-C9	115.0(10)
C11-C10-C9	111.1(8)	C12'-C10'-C9	112.2(11)
C12'-C10'-C11'	109.1(10)	C9-C10'-C11'	112.2(12)
C5-C13-C15	125.2(18)	C5-C13-C14	113.4(16)
C15-C13-C14	109.0(11)	C5-C13'-C15'	102.9(13)
C5-C13'-C14'	108.8(15)	C15'-C13'-C14'	109.0(12)
C21-C16-C17	123.66(17)	C21-C16-N1	118.96(16)
C17-C16-N1	117.37(16)	C16-C17-C18	117.09(18)
C16-C17-C25	121.92(17)	C18-C17-C25	120.96(19)

C19-C18-C17	120.8(2)	C20-C19-C18	120.63(19)
C19-C20-C21	121.35(19)	C16-C21-C20	116.51(18)
C16-C21-C22	121.86(17)	C20-C21-C22	121.62(18)
C21-C22-C23	112.24(19)	C21-C22-C24	110.30(19)
C23-C22-C24	110.5(2)	C17-C25-C26	113.05(19)
C17-C25-C27	110.2(2)	C26-C25-C27	110.6(2)
N4-C28-N3	104.95(14)	N4-C28-S4	127.62(13)
N3-C28-S4	127.42(14)	N3-C29-C30	106.35(14)
N3-C29-S5	126.67(13)	C30-C29-S5	126.96(13)
N4-C30-C29	106.55(14)	N4-C30-S6	126.76(13)
C29-C30-S6	126.60(13)	C36-C31-C32	123.80(18)
C36-C31-N4	117.70(17)	C32-C31-N4	118.47(17)
C31-C32-C33	116.6(2)	C31-C32-C40	122.26(18)
C33-C32-C40	121.1(2)	C34-C33-C32	121.1(2)
C33-C34-C35	120.5(2)	C34-C35-C36	121.5(2)
C31-C36-C35	116.5(2)	C31-C36-C37	122.66(18)
C35-C36-C37	120.9(2)	C38-C37-C36	111.0(2)
C38-C37-C39	111.5(2)	C36-C37-C39	112.0(2)
C32-C40-C41	110.9(2)	C32-C40-C42	111.7(2)
C41-C40-C42	111.6(2)	C48-C43-C44	124.27(17)
C48-C43-N3	117.25(16)	C44-C43-N3	118.48(17)
C45-C44-C43	116.4(2)	C45-C44-C52	121.34(19)
C43-C44-C52	122.25(18)	C44-C45-C46	121.0(2)
C47-C46-C45	121.0(2)	C46-C47-C48	120.9(2)
C43-C48-C47	116.42(19)	C43-C48-C49	122.22(17)
C47-C48-C49	121.34(19)	C51-C49-C48	109.9(2)
C51-C49-C50	110.4(2)	C48-C49-C50	113.18(18)
C53-C52-C44	112.2(2)	C53-C52-C54	110.6(2)
C44-C52-C54	110.5(2)	N5-C55-N6	104.99(13)
N5-C55-S7	127.67(13)	N6-C55-S7	127.34(13)
N5-C56-C57	106.45(14)	N5-C56-S8	127.15(13)
C57-C56-S8	126.36(13)	N6-C57-C56	106.69(14)
N6-C57-S9	126.33(12)	C56-C57-S9	126.90(13)
C63-C58-C59	124.03(16)	C63-C58-N6	117.92(16)
C59-C58-N6	118.01(15)	C60-C59-C58	116.61(17)
C60-C59-C67	121.28(18)	C58-C59-C67	122.05(16)
C59-C60-C61	120.59(19)	C62-C61-C60	121.17(18)
C63-C62-C61	120.96(19)	C58-C63-C62	116.63(18)
C58-C63-C64	122.69(17)	C62-C63-C64	120.68(18)
C66-C64-C63	110.3(2)	C66-C64-C65	112.6(2)
C63-C64-C65	111.3(2)	C69-C67-C59	112.94(19)
C69-C67-C68	109.90(19)	C59-C67-C68	109.96(18)
C75-C70-C71	124.13(17)	C75-C70-N5	117.26(16)
C71-C70-N5	118.60(17)	C70-C71-C72	116.4(2)

C70-C71-C79	121.82(18)	C72-C71-C79	121.77(19)
C73-C72-C71	121.1(2)	C72-C73-C74	120.8(2)
C75-C74-C73	120.9(2)	C70-C75-C74	116.6(2)
C70-C75-C76	122.46(17)	C74-C75-C76	120.9(2)
C77-C76-C78	110.9(2)	C77-C76-C75	111.0(2)
C78-C76-C75	112.71(19)	C80-C79-C71	111.39(19)
C80-C79-C81	110.20(19)	C71-C79-C81	112.3(2)
C88-C82-C83	121.1(3)	C88-C82-C87	121.5(3)
C83-C82-C87	117.4(2)	C84-C83-C82	119.5(2)
C85-C84-C83	121.5(2)	C84-C85-C86	119.1(3)
C87-C86-C85	121.1(3)	C86-C87-C82	121.4(2)
C90-C89-C94	120.0	C90-C89-C95	126.8(10)
C94-C89-C95	112.8(10)	C89-C90-C91	120.0
C92-C91-C90	120.0	C93-C92-C91	120.0
C92-C93-C94	120.0	C93-C94-C89	120.0

Reference:

(1) Stoll, S.; Schweiger, A. EasySpin, a comprehensive software package for spectral simulation and analysis in EPR *J. Magn. Reson.* **2006**, *178*, 42-55.

(2) Wang, Y.; Xie, Y.; Wei, P.; Blair, S. A.; Cui, D.; Johnson, M. K.; Schaefer, H. F., III; Robinson, G. H. A Stable Naked Dithiolene Radical Anion and Synergic THF Ring-Opening *J. Am. Chem. Soc.* **2020**, *142*, 17301-17305.

(3) McPeak, J.; Alexander, D.; Joseph, C.; Eaton, S. S.; Eaton, G. R. Electron Spin Relaxation of Tb^{3+} and Tm^{3+} Ions *Appl. Magn. Reson.* **2020**, *51*, 961-976.

(4) Eaton, G. R.; Eaton, S. S. Relaxation Times of Organic Radicals and Transition Metal Ions In *Distance Measurements in Biological Systems by EPR*; Berliner, L. J., Eaton, G. R., Eaton, S. S., Eds.; Kluwer Academic/Plenum: New York, 2000.

(5) Rajca, A. Organic Diradicals and Polyradicals - from Spin Coupling to Magnetism *Chem. Rev.* **1994**, *94*, 871-893.

(6) Zhang, H.; Pink, M.; Wang, Y.; Rajca, S.; Rajca, A. High-Spin S = 3/2 Ground-State Aminyl Triradicals: Toward High-Spin Oligo-Aza Nanographenes *J. Am. Chem. Soc.* **2022**, *144*, 19576-19591.

(7) Shu, C.; Pink, M.; Junghoefer, T.; Nadler, E.; Rajca, S.; Casu, M. B.; Rajca, A. Synthesis and Thin Films of Thermally Robust Quartet (S = 3/2) Ground State Triradical *J. Am. Chem. Soc.* **2021**, *143*, 5508-5518.
